# In Vitro Oxidative Stress Threatening Maize Pollen Germination and Cytosolic Ca^2+^ Can Be Mitigated by Extracts of Emmer Wheatgrass Biofortified with Selenium

**DOI:** 10.3390/plants11070859

**Published:** 2022-03-24

**Authors:** Alberto Marco Del Pino, Beatrice Falcinelli, Roberto D’Amato, Daniela Businelli, Paolo Benincasa, Carlo Alberto Palmerini

**Affiliations:** Department of Agricultural, Food, and Environmental Sciences, University of Perugia, 06121 Perugia, Italy; alberto.delpino@unipg.it (A.M.D.P.); beatricefalcinelli90@gmail.com (B.F.); roberto.damato@unipg.it (R.D.); daniela.businelli@unipg.it (D.B.); carlo.palmerini@unipg.it (C.A.P.)

**Keywords:** *Triticum dicoccum*, sprouting, salinity, calcium homeostasis, phenolic acid, hydrogen peroxide

## Abstract

In this work, we studied the effects of in vitro oxidative stress applied by H_2_O_2_ to maize pollen germination and cytosolic Ca^2+^, taken as an experimental model to test the biological activity of extracts of emmer (*Triticum turgidum* L. spp. *dicoccum* (Schrank ex Shubler) Thell.) wheatgrass obtained from grains sprouted with distilled water, or salinity (50 mM) or selenium (45 mg L^−1^ of Na_2_SeO_3_). Wheatgrass extracts were obtained in two ways: by direct extraction in methanol, which represented the free phenolic fraction of extracts (Ef), and by residual content after alkaline digestion, which made it possible to obtain extracts with the bound fraction (Eb). Comparative tests on maize pollen were carried out by differently combining H_2_O_2_ and either wheatgrass extracts or pure phenolic acids (4-HO benzoic, caffeic, p-coumaric and salicylic). The cytosolic Ca^2+^ of maize pollen was influenced by either H_2_O_2_ or pure phenolic acids or Ef, but not by Eb. The negative effect of H_2_O_2_ on maize pollen germination and cytosolic Ca^2+^ was mitigated by Ef and, slightly, by Eb. The extent of the biological response of Ef depended on the sprouting conditions (i.e., distilled water, salinity or selenium). The extracts of Se-biofortified wheatgrass were the most effective in counteracting the oxidative stress.

## 1. Introduction

Wheatgrass (1–2 weeks old seedlings of *Graminaceae* species) is recognized as a great source of phytochemicals, i.e., the secondary metabolites of plants having antioxidant activity and related benefits in human health [1,2]. Wheatgrass from *Triticum* species has been extensively studied, and recent trends on healthy diets have been promoting the rediscovery of ancient species, often grown in low-input systems and organic farming [3]. In particular, emmer (*Triticum turgidum* L. spp. *dicoccum* (Schrank ex Schübler) Thell.) has been found to be rich in phenolic compounds, especially phenolic acids (PAs) [4,5], which would also contribute to the abiotic stress tolerance of this species [6,7].

Phytochemicals may have complementary and/or overlapping mechanisms of action [8] and this may explain why the biological activity of wheatgrass is not easy to study and may be different from that expected from each purified molecule it contains. Moreover, phenolic compounds, similarly to other phytochemicals, exist in either free forms or bound forms (covalently conjugated through ester bonds to cell wall components such as cellulose, pectin and polysaccharides), which have different fates and healthy properties after ingestion [9,10].

A body of recent literature demonstrates that the production of phytochemicals in sprouts and wheatgrass of many species can be boosted by appropriate elicitation techniques [1,3,11], with changes also occurring in the proportion between free and bound phenolics. As far as emmer sprouts and wheatgrass are concerned, the application of moderate salt stress was found to increase the total content of phenolic compounds, especially the free ones [6]. On the other hand, bio-fortification with selenium (Se), which has not been tested yet on emmer wheatgrass, has been found to increase phenolic acids and antioxidant activity in sprouts of maize [12] and rice [13], confirming that Se is a powerful protecting agent against abiotic stresses [14].

Although sprouts and wheatgrass of many cereal species have been characterized for the kinds and amounts of phenolic compounds, very few studies have dealt with testing the effects of these matrices on biological systems. In this regard, the measurement of cytosolic Ca^2+^ and germination of maize pollen has proven to be an effective, simple and cheap tool for evaluating the effect of a plant matrix in a biological system [15,16]. In fact, calcium (Ca^2+^) plays an important role in the signal transduction, growth and development of plants [17,18]. Ca^2+^ homeostasis is maintained by keeping the cytosolic ion concentration below 0.1 µM. When cells are stimulated, raising cytosolic Ca^2+^ levels above 200 nM, they activate a molecular signal to trigger downstream responses [19,20,21]. Furthermore, pollen represents a good experimental model, because it can be easily labeled with the FURA 2AM fluorescent probe and kept in suspension, which is a strategy that is not always possible with plant cells. This model is also useful to evaluate the occurrence of oxidative stress, since the increase in reactive oxygen species (ROS) in the cells would alter molecular signals including cytosolic Ca^2+^ [22,23,24]. Oxidative stress is a harmful process that can negatively affect several cellular structures, such as membranes, lipids, proteins, lipoproteins and DNA, and this is the case for both plant [25] and animal cells [26]. With regard to the study of oxidative stress, a body of literature reports the use of hydrogen peroxide (H_2_O_2_) in in vitro experiments. This is because, among the three primary ROS (superoxide anion, hydroxyl radical and hydrogen peroxide), only H_2_O_2_ has a half-life long enough (few seconds) to be used for inducing oxidative stress in vitro [27,28]. Recently, Del Pino et al. [16] demonstrated that extracts of emmer wheatgrass grown with distilled water, or in the presence of salinity (i.e., 50 mM) or Se (45 mg L^−1^), affected cytosolic Ca^2+^ and maize pollen germination. In that case, maize pollen was germinated in optimal conditions and the effects of emmer extracts were evaluated considering only the extracts of free phenolic compounds (i.e., those obtained by extraction with methanol). 

The present work follows up the work by Del Pino et al. [16], by using the same biological model (maize pollen germination and cytosolic Ca^2+^) to test the effect of either free or bound extracts of emmer wheatgrass grown with distilled water, or salinity or selenium, and to evaluate the protective effect against the oxidative stress caused in pollen by the application of H_2_O_2_.

## 2. Results

### 2.1. Total Selenium Content in Extracts of Emmer Wheatgrass

The Se content in Ef_Se_ and Eb_Se_ was 10 and 2.4 times higher, respectively, than in Ef_c_, Ef_s_, Eb_c_ and Eb_s_ (Table 1).

### 2.2. Cytosolic Ca^2+^ of Maize Pollen

**Figure 1:**
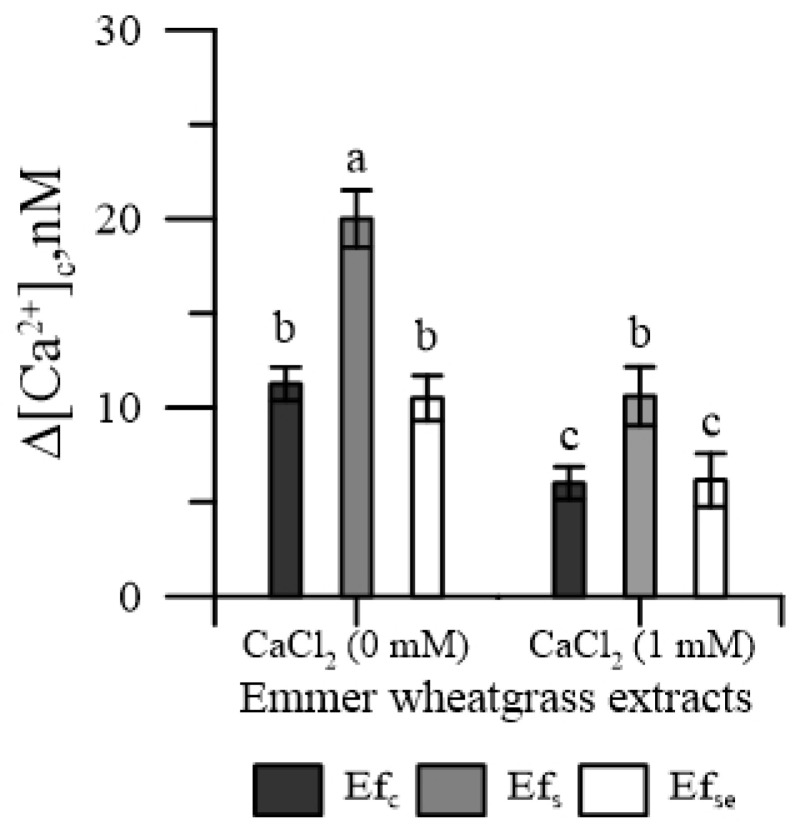
Effects of 50 mg of free extracts (Ef) of emmer wheatgrass obtained with distilled water as a control (Ef_c_), or with NaCl (Ef_s_) 50 mM or Na_2_SeO_3_ 0.45 mg L^−1^ (Ef_Se_), on the cytosolic Ca^2+^ of maize pollen. The measurements were carried out in the absence of Ca^2+^ and in the presence of Ca^2+^ (1 mM CaCl_2_) in the incubation medium. Data are expressed as means ± SEM from four independent tests. Different letters indicate significant differences for *p* < 0.05.

The concentration of cytosolic Ca^2+^ of pollen [Ca^2+^]_cp_ increased with each kind of free extract, both in the absence and in the presence of CaCl_2_ in the incubation medium; however, Ef_s_ caused the greatest increase (Figure 1). On the contrary, none of the bound extracts affected [Ca^2+^]_cp_ (data not shown).

**Figure 2:**
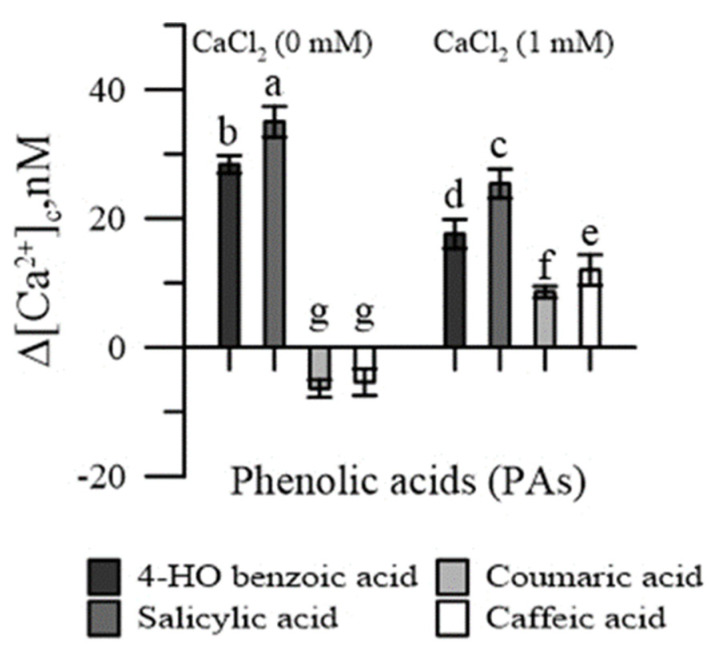
Effects of 4-HO benzoic, caffeic, p-coumaric and salicylic acids on cytosolic Ca^2+^ of maize pollen, in Ca^2+^-free conditions and in the presence of CaCl_2_. Data are expressed as means ± SEM from five independent tests. Different letters indicate significant differences for *p* < 0.05.

In the absence of CaCl_2_ in the incubation medium, salicylic and 4-HO benzoic acids increased [Ca^2+^]_cp_, while coumaric and caffeic acids reduced it (Figure 2). With the addition of CaCl_2_ in the incubation medium, an increase in [Ca^2+^]_cp_ was observed with all PAs, although it was slighter for p-coumaric and caffeic acids.

**Figure 3:**
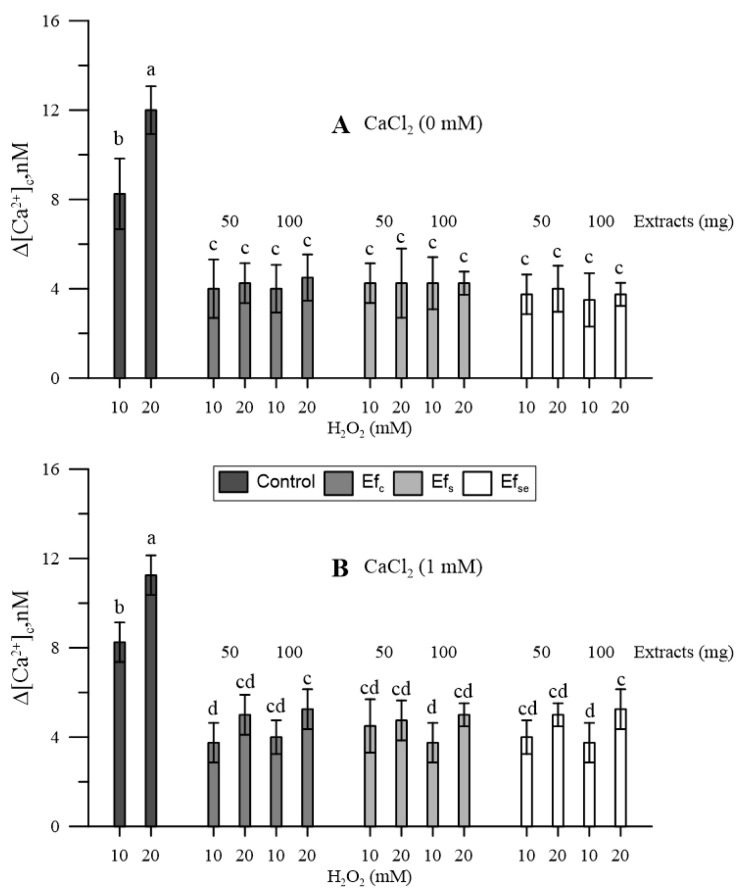
Effects of H_2_O_2_ (10 and 20 mM) on the cytosolic Ca^2+^ of maize pollen pre-treated with 50 or 100 mg of free extracts of emmer wheatgrass grown with distilled water as a control (Ef_c_), or in the presence of salinity as NaCl 50 mM (Ef_s_) or selenium as 45 mg L^−1^ of Na_2_SeO_3_ (Ef_Se_). Maize pollen was (**A**) in the absence or (**B**) in the presence of 1 mM CaCl_2_ in the growing medium. All analyses were performed in triplicate. Data are expressed as means ± SEM from four independent tests. Different letters indicate significant differences for *p* < 0.05.

Hydrogen peroxide increased [Ca^2+^]_cp_; the higher the dose, the higher the increase (Figure 3).

Since the bound extracts had not affected the [Ca^2+^]_cp_, only free extracts (Ef) were tested for their effect against oxidative conditions. All three types of free extracts antagonized the effects of H_2_O_2_ on cytosolic Ca^2+^. The effect did not substantially change in the absence (Figure 3A) or presence (Figure 3B) of CaCl_2_ in the incubation medium, although, in the presence of CaCl_2_, the highest H_2_O_2_ caused further and generally non-significant increases in [Ca^2+^]_cp_ (Figure 3B).

### 2.3. Germination of Maize Pollen

**Figure 4:**
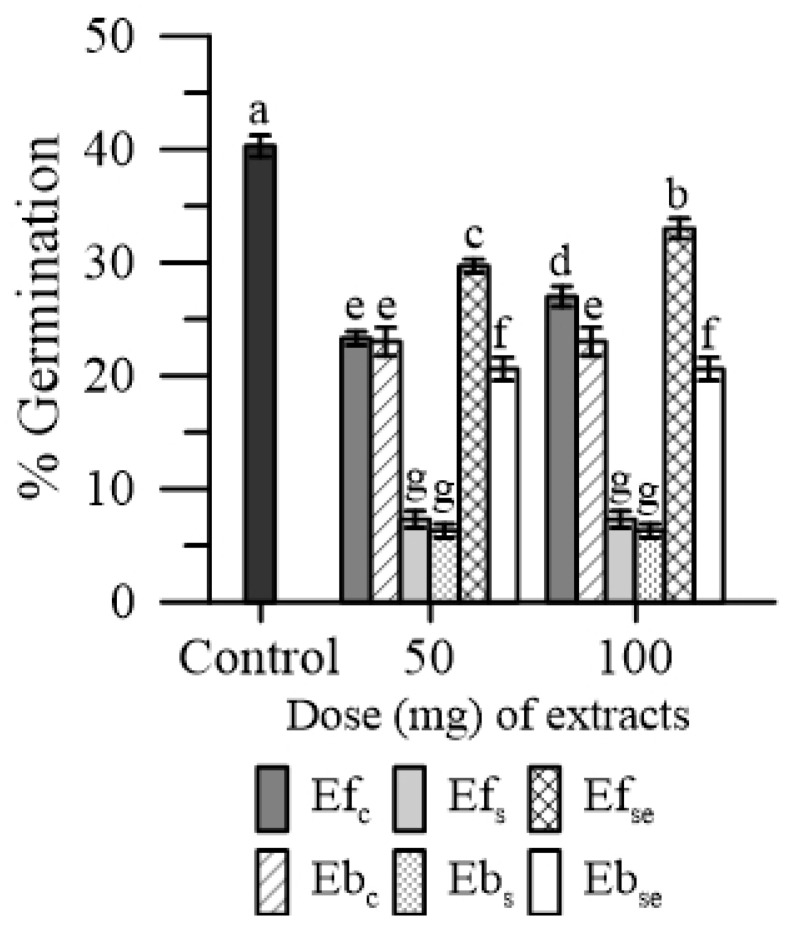
Germination of maize pollen in the presence of 50 or 100 mg of free (Ef) and bound (Eb) extracts of emmer wheatgrass grown with distilled water as a control (Ef_c_, Eb_c_), or in the presence of salinity as NaCl 50 mM (Ef_s_, Eb_s_) or selenium as 45 mg L^−1^ of Na_2_SeO_3_ (Ef_Se_, Eb_Se_). Data are expressed as means ± SEM from five independent tests. Different letters indicate significant differences for *p* < 0.05.

Both Ef and Eb reduced the germination rate of maize pollen. The magnitude of this effect was different between treatments: (Ef_s_ and Eb_s_) > Eb_Se_ > (Ef_c_ and Eb_c_,) > Ef_Se_ (Figure 4).

Considering the Ef and Eb groups, the lowest inhibiting effect on pollen germination was caused by Ef_Se_ (−25%) and Eb_Se_ (−45%). No substantial dose-dependent effect was observed in both Ef and Eb. 

**Figure 5:**
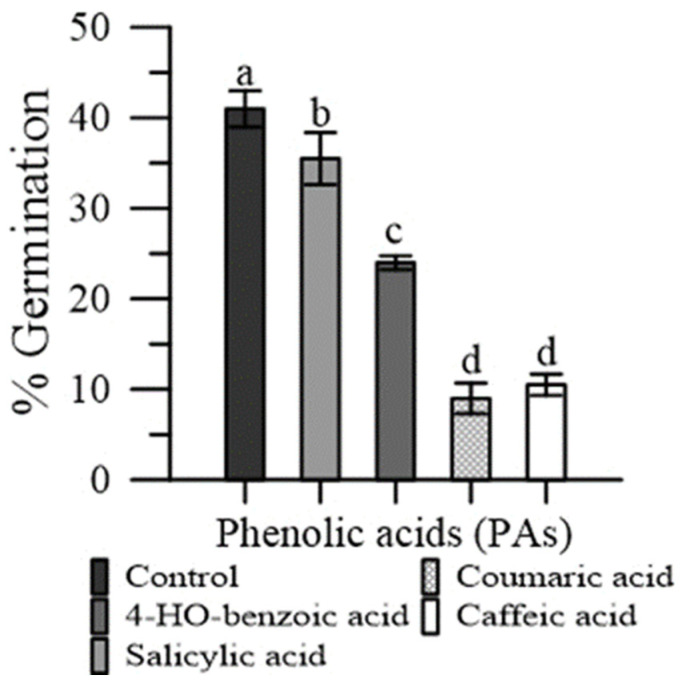
Germination of maize pollen grains in the presence of pure PAs: 4-HO benzoic, caffeic, p-coumaric and salicylic. Data are expressed as means ± SEM from four independent tests. Different letters indicate significant differences for *p* < 0.05.

The p-coumaric and caffeic acid severely depressed pollen germination (−81% and −76%, respectively), while the effect was less marked with 4-HO benzoic acid (−32%) and salicylic acid (−15%) (Figure 5).

**Figure 6:**
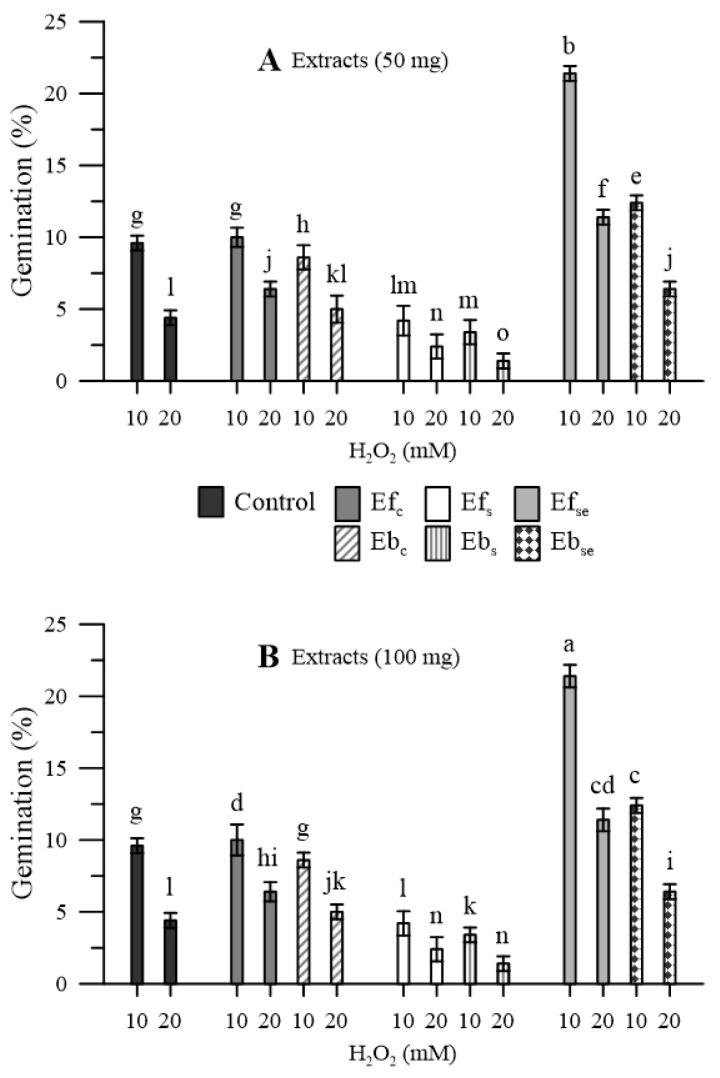
Germination of maize pollen during oxidative stress induced by 10 and 20 mM of H_2_O_2_ as affected by pre-treating the pollen with (**A**) 50 mg or (**B**) 100 mg of free (Ef) and bound (Eb) extracts of emmer wheatgrass grown with distilled water as a control (Ef_c_, Eb_c_) or in the presence of salinity as NaCl 50 mM (Ef_s_, Eb_s_) or selenium as 45 mg L^−1^ of Na_2_SeO_3_ (Ef_Se_, Eb_Se_). Data are expressed as means ± SEM from five independent tests. Different letters indicate significant differences for *p* < 0.05.

In the pollen that was not pre-treated with wheatgrass extracts, the oxidative stress induced, with H_2_O_2_, a marked reduction in the germination rate (−78% and −92% with 10 and 20 mM H_2_O_2_, respectively) (Figure 6). 

A similar situation was observed in the case of pollen pre-treated with extracts of wheatgrass grown in distilled water, while the situation was even worse in the case of wheatgrass grown under salinity. On the contrary, extracts of wheatgrass biofortified with Se allowed an appreciable recovery of the germination percentage, especially in the case of free extracts (Ef_Se_) and milder oxidative stress (10 mM H_2_O_2_). For all three kinds of wheatgrass, free extracts allowed better germination performances that bound extracts. No relevant dose-dependent effects were observed for both free and bound extracts.

## 3. Discussion

The high phytochemical content and antioxidant activity of sprouts and wheatgrass are well documented, whereas the supposed benefits for living organisms have rarely been ascertained. Maize pollen germination and cytosolic Ca^2+^ were used in this work as a model to evaluate the actual effect of emmer wheatgrass and its role in mitigating the oxidative stress caused by H_2_O_2_. Of course, evidence obtained with this model cannot be used to speculate on the effect of wheatgrass on other kind of cells, least of all animal cells. Anyway, the use of this model represents a means to observe integrated effects of the wheatgrass matrix instead of focusing on single compounds. The results obtained in this experiment give rise to some reasoning.

In the absence of oxidative stress, all three types of free extracts of emmer wheatgrass (i.e., either obtained with distilled water, or salinity or selenium) had a negative effect on both the cytosolic Ca^2+^ and germination of maize pollen (Figure 1 and Figure 4), while bound extracts affected only pollen germination. Moreover, the wheatgrass obtained under salinity had the most negative effect (Figure 1).

It is not easy to explain why cytosolic Ca^2+^ was affected only by free extracts, while pollen germination was affected by both free and bound extracts. Assuming that phenolic compounds of extracts might have a role in pollen performances, it is worth pointing out that bound forms are the most represented in emmer wheatgrass [4,5,6] but, differently from free phenolics, they are not readily available to exert their activity. 

Moreover, other undetected phytochemicals could take over pollen germination, as this represents a complex biological event to which many effectors, in addition to cytosolic Ca^2+^, may play a role [22,29,30]. We only examined changes in cytosolic Ca^2+^ because germination is activated by Ca^2+^ signals [29,30]. The specific analytical methods used for the identification of PAs in free and bound extracts could not detect the presence of other active molecules [31]. Finally, the severity of the method used to extract bound phenolics might further explain the low activity of these forms [10]. 

We actually tested four of the most represented PAs in emmer wheatgrass (Figure 2 and Figure 5), two hydroxy-cinnamic (caffeic and p-coumaric) and two hydroxy-benzoic (4-HO benzoic and salicylic) [4,5,6]. All the four PAs perturbed the cytosolic Ca^2+^ homeostasis (Figure 2), the two hydroxybenzoic acids by having an agonist activity, and the two hydroxycinnamic by having a chelating activity. This would suggest that these PAs might be responsible, among other compounds, for the effect of free extracts. It is worth noting that in the presence of external CaCl_2_ 1 mM, the concentration of cytosolic Ca^2+^ was lower than in the absence of external CaCl_2_. This was expected, because the entrance of Ca^2+^ from outside the cell depends on the Ca^2+^ depletion in intracellular stores. Since, in normal conditions, the Ca^2+^ concentration in the cytosol is in the order of nM, while the Ca^2+^ concentration in the extracellular medium was 1 mM, any agent altering the cytosolic ion concentration could affect the molecular mechanisms of homeostasis. In our case, phenolic acids increased cytosolic Ca^2+^ (due to agonist or chelating activity) and depleted the internal stores, causing a higher entrance of the extracellular Ca^2+^ to restore the basal conditions. In particular, the chelating activity of hydroxy-cinnamic acids could explain their higher inhibitory effect on germination. The worsening of pollen performances observed with extracts of wheatgrass grown under salinity (Figure 1 and Figure 4) could be a consequence of the increased content of free phenolics caused by this elicitor, as observed by Stagnari et al. [6] for emmer wheatgrass obtained with 50 mM NaCl in the growing medium. 

In the presence of oxidative stress, all three types of free extracts antagonized the effects of H_2_O_2_ on cytosolic Ca^2+^ (Figure 3). There is a well-known and documented bidirectional relationship between ROS, which can modulate calcium-dependent cellular networks, and calcium signaling, which plays a key role in ROS assembly [22,23,32]. ROS behave as Ca^2+^ agonists, stimulate ion mobilization from internal reserves and activate the entry of Ca^2+^ from the extracellular medium [23,24,33]. Although both H_2_O_2_ and free extracts affected cytosolic Ca^2+^, it is rational to assume that the two agents acted with different molecular mechanisms. Free extracts had a transient effect on the cytosolic Ca^2+^ of the pollen, which was then restored, while H_2_O_2_ caused a prolonged increase in cytosolic Ca^2+^ over time, a depletion of pollen Ca^2+^ reserves and a persistent loss of Ca^2+^ homeostasis. In fact, free extracts mitigated the effects of H_2_O_2_ on cytosolic Ca^2+^, allowing the recovery of Ca^2+^ homeostasis and Ca^2+^ signal function. Bound extracts had no effect. Actually, previous works demonstrated that the highest antioxidant activity in emmer wheatgrass is associated with free phenolics [5], while bound phenolics were found to have low antioxidant activity in both rice [34] and in rapeseed sprouts [35]. 

Only the free extract of the Se-biofortified wheatgrass allowed an appreciable recovery of pollen germination and only at the lowest level of oxidative stress (10 mM H_2_O_2_) (Figure 6). A very slight effect was observed also with bound extracts of Se-biofortified wheatgrass. It is reasonable to assume that the positive effect of the Se-enriched extracts was mainly due to their higher Se contents. In fact, biofortification was successful, as demonstrated by the higher Se content in both the free and bound extracts (Table 1). Selenium is known as a protective agent in oxidative stress [36,37]. The protective effect on the homeostasis of cytosolic Ca^2+^ is one of the positive effects of Se in plants [22,23,28]. Indeed, some studies conducted on maize and olive pollen subjected to oxidative stress reported that Se restored Ca^2+^ homeostasis and improved pollen germination [15,38]. On the other hand, a recent study by Benincasa et al. [12] reported that endogenous Se is a promoter of phenolic compounds and antioxidant activity mainly in combination with other factors, such as salinity. However, the effect of Se-biofortification on phenolic content may be contradictory, depending on the plant species and on the Se dose, as reviewed by D’Amato et al. [14]. Since 100 mg of extract gave no further benefit in the recovery of pollen germination, we can deduce that the amount of Se contained in 50 mg of extract was enough to achieve the maximum effect in terms of mitigation of the stress caused by H_2_O_2_ (saturation effect). Further research with lower doses of Se-biofortified wheatgrass extracts (e.g., including treatments with 10, 20, 30 and 40 mg, besides the treatments with 0 and 50 mg) is needed to ascertain the minimum rate required to achieve the maximum stress mitigation. 

## 4. Materials and Methods

### 4.1. Reagents

Hydrogen peroxide (30% *w*/*v*) and nitric acid (65%, *w*/*v*) were purchased from Suprapur Reagents Merck (Darmstadt, Germany). 4-hydroxybenzoic acid (4-OHBA), caffeic acid (CA), p-coumaric acid (p-CA) and salicylic acid (SA), were purchased from Sigma Aldrich (St. Louis, MO, USA). All standards were prepared as a stock solution at 5 mg mL^−1^ in methanol and stored at −20 °C in the dark. FURA 2-AM (FURA-2-pentakis (acetoxymethyl ester)), Triton X-100 (t-octylphenoxypolyethoxyethanol), EGTA (ethylene glycol-bis (β-aminoethyl ether) −N, N, N′, N′-tetracetic acid), sodium selenite (Na_2_SeO_3_), NaCl, KCl, MgCl_2_, Hepes, dimethyl sulfoxide (DMSO) and CaCl_2_ were purchased from Sigma-Aldrich (St. Louis, MO, USA). Other reagents (reagent grade) were obtained from common commercial sources.

### 4.2. Emmer Wheatgrass Production

Emmer wheatgrass was obtained by germinating grains and growing seedlings with distilled water, or salinity or selenium as described in Del Pino et al. [16]. Briefly, grains of emmer (*Triticum turgidum* L. spp. *dicoccum* (Schrank ex Shubler) Thell., cv. Zefiro) were incubated in plastic trays (20 g per tray) containing sterile cotton and filter paper wetted with distilled water as a control (C), or with a solution containing NaCl 50 mM (S) or 45 mg L^−1^ of Na_2_SeO_3_ (Se), according to a completely randomized block design with four replicates (trays). To maintain the air circulation while preventing dehydration, the trays were covered by a drilled top and then they were incubated in a growth chamber at 20 °C in the dark. After germination, trays were moved in a light–dark regime of 10:14 h, with a light intensity at 200 μmol photons m^−2^ s^−1^. Distilled water was periodically added to trays to restore initial tray weights, considering the change in seedling biomass as negligible, and thus, approximately keeping the initial NaCl and Se concentrations of these treatments [6,7]. Wheatgrass from C treatment was collected 8 days after sowing (DAS), while wheatgrass of S and Se treatments were collected when they reached the same seedling growth stage as in C (9 DAS for S and 11 DAS for Se treatments), because either S or Se slowed seedling growth compared to C. Only shoots were harvested, and replicates of each treatment were re-grouped two by two for the chemical analysis, performed in triplicate. Samples were stored at −20 °C until extraction.

### 4.3. Preparation of Emmer Wheatgrass Extracts

Emmer wheatgrass extracts were obtained, using methanol as a solvent, as described in Del Pino et al. [16] and according to the method of Krygier et al. [39], with slight modifications. Two grams of frozen wheatgrass were mixed with 20 mL of MeOH and homogenized on ice using an Ultraturrax three times, alternating 30 s homogenization and 30 s pause to prevent the material from heating. The solution was then kept in agitation for 24 h and centrifuged at 5000 rpm for 10 min. The supernatant (free fraction) was recovered and evaporated to dryness using a rotary evaporator. The dry extracts were suspended in 2 mL of methanol. This represented the extract of free phenolics (Ef).

The remaining solid residue was mixed with 10 mL of NaOH (5 N) for 1 h and then HCl (5 M) was added until pH = 2. Samples were mixed with 10 mL of ethyl acetate, vortexed and centrifuged at 3000 rpm for 10 min and the supernatant was then recovered (bound fraction). This extraction was performed three times, and the supernatants were pooled and evaporated to dryness using a rotary evaporator. The dry residue was suspended in 2 mL of methanol. This represented the extract of bound phenolics (Eb).

Preliminary tests were carried out on the wheatgrass extracts (Ef and Eb) to evaluate the most suitable concentration for the measurement of cytosolic Ca^2+^ and the germination of maize pollen. Based on the preliminary tests mentioned above, the treatments tested in this study were: free (Ef) and bound (Eb) extracts from wheatgrass grown in distilled water (Ef_c_ and Eb_c_), and with a solution of 50 mM NaCl (Ef_s_ and Eb_s_) or 45 mg L^−1^ Na_2_SeO_3_ (Ef_Se_ and Eb_Se_).

### 4.4. Determination of Total Selenium in Wheatgrass Extracts

Measurements of the total selenium content were performed in all emmer wheatgrass extracts (Ef_c_, Ef_s_, Ef_Se_ and Eb_c_, Eb_s_, Eb_Se_) following the method of D’Amato et al. [40]. Wheatgrass extracts (200 µg) were microwave-digested (ETHOS one high-performance microwave digestion system; Mile-stone Inc., Sorisole, Bergamo, Italy) with 8 mL of ultrapure concentrated nitric acid (65% *w*/*w*) and 2 mL of hydrogen peroxide (30% *w*/*w*). The heating program for the digestion procedure was 30 min at 1000 W and 200 °C. After cooling down, the digests were diluted with water up to 20 mL and passed through 0.45-μm filters. The samples were analyzed by ICP-MS (Agilent 7900, Agilent Technologies, Santa Clara, CA, USA) with an Octopole Reaction System (ORS). Total Se standard solutions were prepared by diluting the corresponding stock solutions (Se standard 1000 mg L^−1^ for AAS TraceCert, 89498, Sigma-Aldrich, Milan, Italy) with HPLC-grade water. Results were expressed as micrograms per kilograms. This method was accurately validated with a recovery test (*n* = 3) by adding a Se standard solution (4 mg L^−1^) into the mixture of Se-enriched sample and nitric acid prior to digestion in tubes and after appropriate dilution.

### 4.5. Measurement of Cytosolic Ca^2+^ with the Addition of Emmer Wheatgrass Extracts, Pure Phenolic Acids and H_2_O_2_ for an Oxidative Stress Induced In Vitro

Cytosolic Ca^2+^ levels were determined spectrofluorometrically using the FURA-2AM probe according to Del Pino et al. [16]. Aliquots (100 mg) of maize pollen, stored in the dark at 5 °C until use, were suspended in 10 mL of PBS and hydrated for 2 days at 25 °C. Hydrated pollens were harvested by centrifugation at 1000 g for 4 min and then resuspended in 2 mL Ca^2+^-free HBSS buffer (120 mM NaCl, 5.0 mM KCl, MgCl_2_ 1 mM, 5 mM glucose, 25 mM Hepes, pH 7.4). Pollen suspensions were incubated in the dark with FURA-2 (2 µL of a 2 mM solution in DMSO) for 120 min, and then centrifuged at 1000 g for 4 min. Pollens were then harvested and suspended in ~10 mL of Ca^2+^-free HBSS containing 0.1 mM EGTA, which was included to rule out or, at least, minimize a potential background due to contaminating ions (so as to obtain a suspension of 1 × 106 pollen granules hydrated per mL).

Fluorescence was measured in a PerkinElmer LS 50 B spectrofluorometer (ex. 340 and 380 nm, em. 510 nm), set with 10 and a 7.5 nm slit widths in the excitation and emission windows, respectively. Fluorometric readings were normally taken after 300–400 s. In detail, the determination of cytosolic Ca^2+^ started after placing the pollen suspension labelled with FURA 2AM in the cuvette and lasted for 100 s. 

After determining the basal cytosolic Ca^2+^ content of the pollen, the following agents were added, singularly or in different combinations according to specific purposes. In detail, aliquots (50 mg) of each of the three free (Ef_c_, Ef_s_, Ef_Se_) and three bound (Eb_c_, Eb_s_, Eb_Se_) extracts, and aliquots of pure phenolic acids (0.250 mg of 4-HO benzoic, caffeic, p-coumaric, and salicylic acid) were used in the assay to determine the effect of wheatgrass extract and of most representative wheatgrass PAs on the cytosolic Ca^2+^ of maize pollen labelled with the FURA 2AM fluorescent probe. The measurements were carried out in the absence of Ca^2+^ (Ca^2+^-free) and in the presence of Ca^2+^ (1 mM CaCl_2_) in the incubation medium.

Oxidative stress was induced in vitro with hydrogen peroxide at 10 and 20 mM, and in the absence of Ca^2+^ (Ca^2+^-free) or presence of Ca^2+^ (1 mM CaCl_2_) in the incubation medium, and in the absence or presence of two doses (50 and 100 mg) of each of the three free extracts. 

After the addition of each test agent, changes in cytosolic calcium were monitored for another 200–300 s. Cytosolic calcium concentrations of pollen ([Ca^2+^]_cp_) were calculated following Grynkiewicz et al. [41]. The extent of the determined variations was expressed as Δ[Ca^2+^]_cp_, nM.

### 4.6. Germination of Maize Pollen Grains with the Addition of Emmer Wheatgrass Extracts, Pure Phenolic Acids and H_2_O_2_ for an Oxidative Stress Induced In Vitro

Fresh pollen samples from each plot were hydrated in a humid chamber at room temperature for 30 min [42], and then transferred to 6-well culture Corning plates (1 mg of pollen per plate) containing 3 mL of an agar-solidified growing medium composed of 1.2% agar, 10%, sucrose, 0.03% boric acid and 0.15% calcium chloride (pH 5.5) [43]. Pollen suspensions were incubated for 24–48 h in a growth chamber at 27 °C with gentle shaking to ensure homogeneous distribution of the samples in the wells.

Two doses (50 or 100 mg) of free and bound (E_f_ and E_b_) extracts, and aliquots of pure phenolic acids (0.250 mg of 4-HO benzoic, caffeic, p-coumaric and salicylic acid) were applied to test, in vitro, the germination on maize pollen grains. 

Oxidative stress was induced in vitro with hydrogen peroxide at 10 and 20 mM, and in the absence or presence of two doses (50 and 100 mg) of each of the three free or bound extracts. 

Germinated and non-germinated pollen grains were counted under a 10x magnification microscope. Germination rates were calculated based on three replicates, each of which consisted of 100 grains. Germination of grains was confirmed when the pollen tube had grown longer than the grain’s diameter [43]. 

### 4.7. Statistical Analysis

Statistical evaluations were performed using the OriginPro software [44]. Variance assessments included homogeneity analysis using the Levene test and normality analysis using the D’Agostino–Pearson test. Significance of differences was assessed by the Fisher’s least significant differences test, after the analysis of variance according to the randomized complete split-plot with five (Figure 2, Figure 4 and Figure 6) or four replicates (Figure 1 and Figure 3), and with randomized complete one-way design with four replicates for Figure 5. The results obtained are expressed as mean values ± standard error of the mean (SEM). Differences with *p* < 0.05 were considered statistically significant.

## 5. Conclusions

Maize pollen germination and cytosolic Ca^2+^ were confirmed to be a good model to test the integrated effects of the wheatgrass matrix. Cytosolic Ca^2+^ was affected only by free extracts of emmer wheatgrass, while the germination of maize pollen was affected by both free and bound extracts. Based on the effects observed by applying pure phenolic acids, it is reasonable to assume that these compounds may partly contribute to the effects of the extracts, but other compounds are likely involved, which deserve to be further studied. The extracts, mainly the free ones, were able to counteract the perturbation of Ca^2+^ homeostasis caused by H_2_O_2_ and to slightly mitigate its depressive effect on pollen germination. The mitigation effect depended on the wheatgrass growing conditions (i.e., distilled water, salinity or selenium). The best results were obtained with Se-biofortified wheatgrass, likely due to the Se itself rather than to its boosting effect on phenolic compounds.

## Figures and Tables

**Table 1:** Total selenium content in the free and bound extracts of emmer wheatgrass grown with distilled water as a control (Ef_c_), or in the presence of salinity as NaCl 50 mM (Ef_s_) or selenium as 45 mg L^−1^ of Na_2_SeO_3_ (Ef_Se_). All analyses were performed in triplicate.

Free Extracts	Se Content (ppb)	Bound Extracts	Se Content (ppb)
Efc	108 ± 10	Ebc	105 ± 8
Efs	111 ± 13	Ebs	109 ± 11
EfSe	1155 ± 25	EbSe	240 ± 15

## Data Availability

The data that support the findings of this study are available from the last author (C.A.P.) on reasonable request.
